# Sketches of the Knowledge of Teeth.—The Origin of Medical Science

**Published:** 1856-04

**Authors:** George Hayes

**Affiliations:** Dentist, Conduit Street, Hanover Square, London.


### ARTICLE III.

*Sketches of the Knowledge of Teeth.—The Origin of Medical Science.* By GEORGE HAYES, Esq., Dentist, Conduit Street, Hanover Square, London.

THE history of dental science is, like any other, interesting on account of showing where human ken and knowledge began, and where they have led hitherto. It exhibits to us the many phases of human investigation, thought and industry, to arrive at certain aims and scopes demanded by, and connected with, the general exigencies of the times. But the history of any department of medical science exhibits also that melancholy truth, that while medical science has expanded and advanced, that *high* and *sublime object* thereof—*man*, has deteriorated. The science of dentistry, hardly touched by the writers of antiquity, has grown up to an extraordinary bulk, which, however, like other branches of pathology and therapeutics, may now require to be involuted and contracted, by the aid of hygeistic principles. Dental science in fine has originated from such slender sources, and has been so completely identified with the other branches of surgery and medicine, by the writers of old, that we cannot dilate on it without alluding to the former.

The origin of medical appliances and science—appertaining to times anti-historical, cannot be looked for in history, but must be traced by analogy and pshycological and physiological induction. Man in his rudest state must soon have found, that if any external accident, a fall or hurt has injured him, the approach to the fire, or in other cases the contact with cold water, &c., would relieve him. And thus, he must successively have arrived at the conviction, that there exist certain external appliances and remedies, which will allay some or other morbid state of his body. As in the lapse of time, seers and prophets or priests took hold of man's affairs, both religious and social—medicine also must have necessarily become one of their studies and occupations; a field, which once entered upon, became boundless, as most other pursuits of the human mind. Whether we may derive the origin of civilization and science from the Egyptians or Hebrews, we find that in both countries *it were* the priests, who first practiced the art of healing, and with the former nation *dentistry* is especially mentioned as one of its departments. Herodotus, who had travelled through most parts of the then known world, (500 B. C.,) but who, for his descriptions, availed himself often of much older dates, as is the case with the narrations of the Egyptian priests—has left us the following interesting passage: "The art of medicine is thus divided amongst them, (the Egyptians,) each physician applies himself to one disease only and not more. All places abound in physicians; some physicians are for the eyes, others for the head, others for the teeth, and others for internal disorders."

Herod. ii, 84. Although we may almost say, that the objects of Egyptian antiquity, which have reached us, are numberless; still, with the exception of some doubtful *sondes*, no surgical instrument has been discovered yet; which, if they were of steel, may be easily accounted for. Still, the following passage, derived also from Herodotus, clearly proves that surgical instruments of some kind were in use even in the remotest antiquity. "It happened not long after, that Darius, in leaping from his horse while hunting, twisted his foot—and at first thinking he had about him those of the Egyptians who had the first reputation for skill in the healing art, he made use of their assistance. But they, by twisting the foot, made the evil worse. As he still continued in a bad state, some one had before heard at Sardis of the skill of Democedes, the Crotonian, made it known to Darius. Upon which, when Darius put himself under his care, he, in a little time, restored him to his health. This Democedes visited Polycrates, after having left Crotona and went to Ægina; having settled there, though he was unprovided with means and had none of the instruments (!) necessary for the exercise of his art, he surpassed the most skillful of their physicians." Herod. iii, 129, 131.

It is also difficult to pronounce on the priority of the custom, prevalent both in Egypt and Greece, to suspend votive tablets in the temples; and to inscribe columns with medical and other precepts. In Egypt, the sciences, said to have been discovered by Thouth, were early collected into a book of papyrus entitled *Embre*, into which the rules of medical science were consigned. In the time of Jamblichus, the Egyptian priests possessed medical books ascribed to Hermes, of which some treated of surgical instruments.

As the sacred books of the Hebrews are neither numerous nor of extensive scope, they afford few glances at the state of their medical knowledge, except that it was very restricted and crude, as perhaps the maladies of those times were also few and of a milder character. The passage moreover from Gen. l, 2, "and Joseph ordered his physicians (!) to preserve his father with aromatics," might indicate that the Hebrews had derived their medical knowledge also from that, even then, highly cultivated nation, the Egyptians. The Holy Writ informs us, that the Hebrews used lotions, frictions, pigments and unctions, cataplasms of figs and a certain sort of *gum*, and employed ligatures for fractured limbs—remedies common with most infant people. But it is hardly to be supposed, that they performed *operations*, as their dread of dead bodies was even supported by an especial law, and made them averse to any procedure where life and death are so contiguous.

With the Greeks, the inventor (*god*) of medicine was Esculapius, a son of the sun, and his sons Podalire and Machaon were counted by Homer amongst the heroes who besieged Troy. In Greece (as well as Egypt) it were the *priests* who attended to the ailments of man, and the temples destined for it were called Æsculapions, mostly situated in high and healthy localities, surrounded by shady groves. One of the most ancient was that of Cnidos, and thence came forth the first known *book* on medicine; and one of the most important works of Hippocrates, (460, B. C.,) is a refutation of these knidian gnomes.

The medical men (Æsclepiades) of those times formed a corporation, which, at the beginning, had the exclusive privilege of medical practice in the Æsculapions and outwardly. They also had the obligation of recording on tablets, the principal cases and their treatment, which were then brought into a more digested form and called *knidian sentences.* The exercise of the healing art, by priests, in fine, implies an admonition on the exhibition of that *humanity* and conscientiousness, which constitute the highest qualities, especially of the operative surgeon.

In the earliest times, the precepts and operation of the healing art were kept exclusive—caste-like, handed down from father to son, &c. Besides the then generally rare use of scriptorial art, Galen (lib. ii,) expressly states, why at first nothing on the art of healing was written down: "They learnt," says that ancient author, "from their youth the art of anatomy from their fathers, and considered it unnecessary to write thereon, as upon the art of writing itself. In the course of time, however, the art was not only propagated by inheritors to sons and other relations, who had to practice it from their very infancy; but it was communicated also to strangers, and to such men as were distinguished by certain qualities." What Galen says here of anatomy may be as well applied to all other departments of the healing art. At first, however, the precepts and operations were kept *secret*, and only communicated under the seal of secrecy. Thus, in Lucian, we find the following words put into the mouth of a priest of medicine: "A sacred mysterious oath binds me: I am commanded by the last words of my dying father."* In that fine piece of composition, called *the law of Hippocrates*, is to be found the following passage: "holy things are communicated only to the initiated, but they must not be trusted to the profane, before they have been initiated into the orgies of the science." From this mysterious character of medicine sprung up those beautiful axioms, which would do honor to the best amongst us, even in this refined age:

"If you love men—you will love your art."

"The physician, who is also a philosopher, is to be reckoned with the gods."

The Æsculapions were, moreover, not only temples and places of treatment for the ailing, but became necessary also schools of the medical art. Such were those of Cyrene, Rhodes, Cnidus and Cos, about 500, B. C. But it is difficult to fix their precise date of antiquity. The way in which medical writings arose out of their temples, were manifold. The sick, who went there to be treated, assumed the custom of leaving behind some written memento of their thankfulness towards the gods, and which characterized and described the malady, of which they had been cured; or they brought thank offerings to the gods, made donations to the priests, or presented some sacred vessel to the temple. Or they had the parts at which they had suffered, made of gold, silver, ivory, &c., and also exhibited them in the temples. Or the names of the patients, their maladies and the remedies resorted to were inscribed on metal tablets or on columns. It became also customary to inscribe there the

*Lucian. Tragopadagr., v, 270.

preparation of some other remedy. These tablets were probably soon annotated by the priest-physicians, and long before Hippocrates, there existed a collection of semiotic precepts, known by the name of the "caik prenotions."

The Cnidion school divided the maladies into a great number of species, seven maladies of the bile, twelve of the bladder, &c. It would, however, appear, from a passage of Plato, (lib. iii,) that the Æsclapiades did not prescribe for anything but wounds and *acute* diseases; as Plato reproaches Herodius, of Sylimbria, for prolonging the life of sickly persons, and making them thus linger by a long malady, rather than let nature free them of their sufferings by death. But Herodius employed chiefly *gymnastics* as a curative agent, and thus another radius was added to the sphere of Greek medical art.

The preceding may be called the *first period* of medical science—as an esoteric knowledge preserved amongst a caste or family. But the approach of the fifth century before Christ was an epoch of *considerable revolution* in the vast domain of the human mind, in which, medical science had its appropriate share. It was the era of Socrates, Plato, Anaxagoras, Empedocles, Herodotus and Thucydides, as well as that of Phidias, Sophocles and Euripides. It was the epoch of the emancipation of medical science, and what had been before *theology* became now *anthropology*—a change, on the advantage of which we do not wish here to dilate. Still, out of the temples learned men began now to occupy themselves with the study of *nature*, alloying it with all the fancy and sublimity of that philosophy peculiar to the Greek sages. Such were Melissus, Domenides, Empedocles, &c., whose writings have, however, only reached us in scanty fragments. But out of this novel tendency arose the medical school of Croton, in Italy—the first which seemed unconnected with any Æsclapion or the Æsclapiades; and Herodotus, who lived not far off, inform us, that it was the most celebrated of those times.

The mind of man thus cultured became prepared for the appearance of *Hippocrates*, one of the greatest medical men who ever existed. It is in his writings where first some positive facts on the maladies of the *teeth* are to be found, and which we have (we think) collected for the first time. It was also for this reason, that we have prefaced some data of the earliest Greek medical history; as else, the mentioning of Hippocrates and his knowledge of the human teeth, would stand solitary and unconnected. In appreciating the opinions of this great man, we must judge him in connection with the very imperfect knowledge of anatomy and physiology of those times, now twenty-three centuries ago. He says: "There is a glutinous increment from the bones of the head and the jaws, of which the fatty part is dried up, and the teeth are made harder than the other bones, because there is nothing cold in them." "In the fœtus they are nourished by the food of the mother and after birth by the milk which the infant sucks from the breast." He alludes to the *wise teeth,* and holds the opinion, that to have a greater number of teeth is a sign of longevity.

The following therapeutic observations are very interesting: "With a child suffering under phagedenic affection the upper and lower teeth fell out; as the bone (jaw) had become hollow. The losing of a bone of the palate causes the lowering of the nose in its middle; the losing of the upper front teeth causes the flattening of the base of the nose. The fifth tooth counted from those in front; four roots united, two by two to each of the neighboring teeth; and all turned inside by their points. The third tooth is more subjected to suppuration, than all the rest; and the thick flux of the nostrils as well as the pains of the temples come chiefly from that tooth. This tooth is much subject to caries, especially the fifth. This tooth had a tuberosity in the middle, and two in front; a small protuberance on the inner side, at the side of the two others, was first subject to caries. The seventh has a single large root pointed. With the boy of Atherata the lower tooth to the left and the upper to the right, the right ear suppurated, at the period when he did not suffer any more." Epid. iv, 19. "The wife of Aspasius had violent tooth-ache, the jaws swelled, having used a callert oxium of castoreum and pepper, she was relieved." Epid. v, 67. "Melesander, the gums being affected, swollen and very painful, he was bled on the arm; Egyptian alum helps at the outset." Epid. v, 69.

"At Cardia, the child of Metrodore, in consequence of toothache, had a sphacelus of the jaw overgrowing flesh on the gums, the suppuration was middling, the molar teeth and the jaw fell (out.)" Epid. v, 100.

We have reproduced these curious extracts as we have found them, although some are not quite clear—the further explanation of which, we leave to other hands. What must strike evey one is, that we find here mentioned diseases of the teeth of a severe character. Hippocrates does not state that *extraction* had been resorted to. This anomaly may be partly explained by the fact, that Hippocrates (or his compilers) does, in the main, only speak of the symptom and issues of maladies, without stating what remedies and expedients were resorted to. Still, it is interesting to inquire about the first glimpses of anatomy and its natural consequences (!)—surgical operations.

With the Egyptians the dread of the dead was so great, that the person who made the incision for introducing the *aromas* into a dead body, had to run away after he had made it, &c. But it seems, that when the Ptolemies had succeeded to the Pharaohs—they, as universal Mœcenses of science and art, were also induced to patronize anatomy; and it is especially recorded, that Erasistratus and Herophylus, (400, B. C.,) obtained through their favor the bodies of malefactors for dissection; and it is even said, that they dissected the bodies of the *living*. However this may be, a practice of anatomical inquiry must have led to the only *real* and *genuine* knowledge of the fabric of the human body, and thence to the conviction, that certain ailments and defects may be removed by anatomical procedures. Strange to say, however, that it is the same Herophylus and another physician Heraclidus of Tarent, who are recorded as dental operators. The passage alluded to runs thus: "Herophylus and Heraclidus of Tarent have recorded a novel mode of extracting teeth. Because it is said, that Erasistratus had deposited some leaden *odontogogam* which we should call a tooth-drawer, (denti-ducum,) at Delphos in the temple of Apollo, as a matter of show, and to prove, that the teeth ought to be removed that are loose or relaxed or for which a leaden instrument (*ferementum*) will suffice. How, however, carious (*exesi*) or loose teeth are to be removed, I have spoken of in my books of medicinal responsions."* A variety of corollaries may be deduced from this passage, amongst which that, that many teeth do not require the forcible application of steel—may be consoling to a number of nervous and timid patients.

The mentioning of Egyptian alum as a dental remedy by Hippocrates, is also very curious, as we come to know, that even now this salt is considered a secret remedy against tooth-ache amongst the Jews; and as their talmudic traditions remount to the times of Moses, it would be curious to think, that even the knowledge of this simple remedy should have remained with them since times immemorial.

With Hippocrates, in fine, we may consider the *first epoch* of dental medicine to be concluded—one of extreme simplicity, if not of limitation.

* Cœluis Aurelinus de Morbis atcutis aque chronicis. Amstel. 1709. 4to.

